# Behavior of non-prismatic RC beams with conventional steel and green GFRP rebars for sustainable infrastructure

**DOI:** 10.1038/s41598-023-41467-w

**Published:** 2023-09-21

**Authors:** Suniti Suparp, Inamullah Khan, Ali Ejaz, Kaffayatullah Khan, Uruya Weesakul, Qudeer Hussain, Panumas Saingam

**Affiliations:** 1 Department of Civil and Environmental Engineering, Faculty of Engineering, Srinakharinwirot University, Nakhonnayok, 26120 Thailand; 2 National Institute of Transportation, National University of Sciences and Technology (NUST), Islamabad, Pakistan; 3 Department of Civil and Environmental Engineering, College of Engineering, King Faisal University, P.O. Box 380, 31982 Al-Hofuf, Al-Ahsa Kingdom of Saudi Arabia; 4 Thammasat University Research Unit in Climate Change and Sustainability, Department of Civil Engineering, Faculty of Engineering, Thammasat School of Engineering, Thammasat University, Pathumthani, 12120 Thailand; 5 Dr. House Consultants Co. Ltd., Bangkok, Thailand; 6 Department of Civil Engineering, School of Engineering, King Mongkut’s Institute of Technology Ladkrabang, Bangkok, 10520 Thailand

**Keywords:** Civil engineering, Composites

## Abstract

This study presents an experimental and finite element analysis of reinforced concrete beams with solid, hollow, prismatic, or non-prismatic sections. In the first part, a total of six beams were tested under four-point monotonic bending. The test matrix was designed to provide a comparison of structural behavior between prismatic solid and hollow section beams, prismatic solid and non-prismatic solid section beams, and prismatic hollow and non-prismatic hollow section beams. The intensity of shear was maximum in the case of prismatic section beams. The inclusion of a tapered section lowered the demand for shear. In the second part, Nonlinear Finite Element Modeling was performed by using ATENA. The adopted modeling strategy resulted in close agreement with experimental crack patterns at ultimate failure. However, the ultimate failure loads predicted by nonlinear modeling were generally higher than their corresponding experimental results. Whereas in the last part, the developed models were further extended to investigate the effect of the strength of concrete and ratio of longitudinal steel bars on the ultimate load-carrying capacity and cracking behavior of the reinforced concrete beams with solid, hollow, prismatic, or non-prismatic sections. The ultimate loads for each beam predicted by the model were found to be in close agreement with experimental results. Nonlinear modeling was further extended to assess the effects of concrete strength and longitudinal reinforcement ratio on failure patterns and ultimate loads. The parametric study involved beams reinforced with glass fiber-reinforced polymer (GFRP) bars against shear and flexural failure. In terms of ultimate load capacities, diagonal cracking, and flexural cracking, beams strengthened with GFRP bars demonstrated comparable performance to the beams strengthened with steel bars.

## Introduction

Bridges are a vital part of the infrastructure of any country. Bridges with long spans are often constructed in modern construction works. One of the key concerns in long-span bridges is related to self-weight. The use of non-prismatic sections along the span can significantly lower the self-weight of the structure. In addition, novel aesthetic designs are possible from an architectural standpoint^1–3^. The use of non-prismatic beams can help decrease the clear ceiling heights: a desirable feature from the cost perspective. The use of hollow-section members has also been a practice to reduce the self-weight, with their applications in almost every kind of structure^4–7^. Reinforced concrete (RC) beams with hollow sections provide a viable solution for the passage of sewage or transmission lines^8–10^. The behavior of RC beams with non-prismatic or hollow sections has been investigated by numerous researchers separately. Abbas et al.^9^ studied the behavior of high-strength concrete hollow beams strengthened with steel fibers. Vijayakumar and Madhavi^11^ tested RC beams with an 80 mm circular opening along the neutral axis. The cracking, as well as the ultimate load of hollow beams without hybrid fiber strengthening, were found to be lower than those of the strengthened beams. El-kassas et al.^12^ RC deep beams with square and circular openings. In addition, the location of openings was varied, i.e., within the compression or tension zones. It was concluded that the size of openings had an adverse effect on the ultimate load capacity. Abbas et al.^13^ tested RC beams with square holes having side lengths of 60 mm, 80 mm, or 100 mm. The ductility of beams with size reductions of 16.0% and 28.4% was found to be higher than the ductility of corresponding solid beams, whereas no noticeable difference was observed when the size reduction was 44.4%. Elamary et al.^14^ tested four beams: one with a solid section and three beams with different opening sizes as 3%, 7%, and 10% of the gross cross-sectional area. It was concluded that an opening size of 10% of the gross cross-sectional area did not significantly lower the ultimate capacity.

According to Al-Ahmed et al.^15^, the current design codes of ACI^16^ and BS^17^ do not provide specific guidance for the design of non-prismatic beams due to insufficient knowledge of their failure mechanism. Tena-Colunga et al.^18^ tested non-prismatic (haunched) beams with and without shear reinforcement. The haunched beams were found to exhibit different behavior than prismatic beams, with haunched beams favoring arching action. Caldentey et al.^19^ tested eight slender beams: four with prismatic sections and four with variable depth. The type of loading and tapered geometry were found to significantly degrade the shear capacity. Qissab and Salman^20^ found that increasing the tapered angle from $7^\circ$ to $12^\circ$ increased the ultimate shear capacity by 45.5%. Other studies enhanced the shear capacity of non-prismatic beams with fiber wraps or by using high concrete strength^21–23^. It has been shown that several studies exist in the literature focusing on hollow or non-prismatic beams separately. However, experimental work on non-prismatic beams with hollow sections is limited. Dawood and Nabbat^1^ tested twelve RC non-prismatic beams with and without openings. It was found that the presence of openings in non-prismatic beams reduced the load capacity up to 10.8% when compared to the capacity of the corresponding beam with a solid section. Al-Maliki^24^ tested five RC non-prismatic hollow section beams. It was found that the presence of a circular opening reduced the ultimate capacity by up to 53% and increased ultimate deflection by up to 40% when compared to the response of solid non-prismatic beams. Alshimmeri et al.^25^ conducted a numerical study by using the commercial finite element software ANSYS. The numerical results revealed a reduction of more than 50% in load capacity when the solid section of the non-prismatic beam was replaced by a hollow section. It is clear from the previous discussion that experimental work on the behavior of non-prismatic beams with hollow sections is limited. Recognizing this, the present study aims to investigate the role of openings in non-prismatic beams. In addition, the present study also investigates the behavior of solid section beams with and without tapered sections. Nonlinear modeling was performed using the software ATENA to model the beams considering internal openings and tapered sections. Additionally, the nonlinear FEM was extended to study the effect of concrete strength and longitudinal bar diameter on failure patterns. The ultimate capacity of RC members can either be predicted by using empirical expressions^26^ or by using FEM^27–30^. In addition, real-time monitoring systems are also deployed to detect cracks and deterioration of RC members^31,32^.

Recently, the use of GFRP bars in structural applications has gained significant importance, attributed to their substantial resistance to corrosion as compared to conventional steel bars^33,34^ and steel corrosion has a negative impact on the durability of RC structures^35^. GFRP, in the form of external wraps, has been successfully utilized in the strengthening of RC members^36–38^. In addition, GFRP bars offer a significant ultimate strain that could be enough to alert before failure^39,40^. Further, FRP bars have proved to be a feasible alternative to steel bars in prismatic beams^41^. Thus, a viable solution against the deteriorating deicing solutions in terms of sustainability could be obtained using GFRP bars. An effort was made to assess the behavior of non-prismatic concrete beams reinforced by GFRP bars with the use of the developed FEM models. The effect of the diameter of flexural GFRP bars was varied to study its effect on the ultimate load capacity and cracking patterns of prismatic and non-prismatic beams with solid and hollow sections. Additionally, a comparison is made on cracking patterns of steel reinforced and GFRP reinforced beams by using FEM results.

## Experimental program

### Test matrix and analysis program

**Figure 1:**
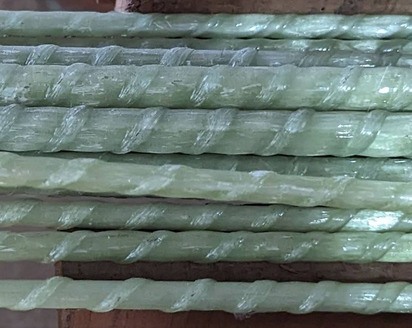
Typical GFRP bars.

The present study investigated the structural response of beams with non-prismatic, hollow, or a combination of both sections. In the first part, a total of six beams were tested. The details of the tested beams are presented in Table 1. Three beams, i.e., P-S-01, NP-S-02, and NP-S-03, were constructed with solid sections. The remaining three beams, i.e., P-H-04, NP-H-05, and NP-H-06, were constructed with hollow sections. P-S-01 and P-H-04 were constructed with prismatic sections. Beams NP-S-02, NP-S-03, NP-H-05, and NP-H-06 incorporated non-prismatic sections along their spans. However, the length of the non-prismatic span in specimens NP-S-02 and NP-H-05 was smaller than that of the specimens NP-S-03 and NP-H-06. In the second part, the experimentally tested beams (Table 1) were analyzed in computer program ATENA (“Finite element modeling”). Whereas in the last part, parametric finite element modeling was performed to study the effect of the strength of concrete and longitudinal reinforcement ratio on the ultimate load-carrying capacity and cracking behavior of prismatic and non-prismatic RC beams with solid and hollow sections. The developed finite element models were further extended, and a total number of 12 RC beams were modeled in ATENA. The details of parametric RC beams are shown in Table 2. In beams P-S-01-HSC, NP-S-02-HSC, NP-S-03-HSC, P-H-04-HSC, NP-H-05-HSC, and NP-H-06-HSC strength of concrete was increased to 30 MPa to study the effect of strength of concrete on the ultimate load carrying capacity and cracking behavior of prismatic and non-prismatic RC beams with solid and hollow section. Whereas in beams P-S-01-HRR, NP-S-02-HRR, NP-S-03-HRR, P-H-04-HRR, NP-H-05- HRR, and NP-H-06- HRR, two deformed bars of diameter 25 mm were used to assess the effect of high reinforcement ratio. In the following section, the finite element analysis results of these beams were compared with the beams P-S-01, NP-S-02, NP-S-03, P-H-04, NP-H-05, and NP-H-06. Further, finite element modeling was extended to study the behavior of non-prismatic solid and hollow beams reinforced with glass fiber-reinforced polymer (GFRP) bars. It is important to note that the type of bars for the shear and flexural reinforcement were GFRP bars. Two sizes of GFRP bars were considered for the bottom longitudinal reinforcement, i.e., diameters of 16 mm and 25 mm, whereas the concrete strength was kept at 15 MPa. Typical GFRP bars are shown in Fig. 1.

**Table 1:** Details of the beam specimen of experimental program.

Type of beams	Shape	Type of prismatic section	Section	Total length of non-prismatic section (mm)
P-S-01	Prismatic	–	Solid	–
NP-S-02	Non-prismatic	Type-1	Solid	400
NP-S-03	Non-prismatic	Type-2	Solid	800
P-H-04	Prismatic	–	Hollow	–
NP-H-05	Non-prismatic	Type-1	Hollow	400
NP-H-06	Non-prismatic	Type-2	Hollow	800

**Table 2:** Details of the beam specimens of parametric study.

Flexural reinforcement					
Steel			GFRP		
Beams ID	Concrete strength (MPa)	Bar Size	Beams ID	Concrete strength (MPa)	Bar size
P-S-01-HSC	30	2DB16	P-S-01-GFRP16	15	2DB16
NP-S-02-HSC	30	2DB16	NP-S-02-GFRP16	15	2DB16
NP-S-03-HSC	30	2DB16	NP-S-03-GFRP16	15	2DB16
P-H-04-HSC	30	2DB16	P-H-04-GFRP16	15	2DB16
NP-H-05-HSC	30	2DB16	NP-H-05-GFRP16	15	2DB16
NP-H-06-HSC	30	2DB16	NP-H-06-GFRP16	15	2DB16
P-S-01-HRR	15	2DB25	P-S-01-GFRP25	15	2DB25
NP-S-02-HRR	15	2DB25	NP-S-02-GFRP25	15	2DB25
NP-S-03-HRR	15	2DB25	NP-S-03-GFRP25	15	2DB25
P-H-04-HRR	15	2DB25	P-H-04-GFRP25	15	2DB25
NP-H-05-HRR	15	2DB25	NP-H-05-GFRP25	15	2DB25
NP-H-06- HRR	15	2DB25	NP-H-06-GFRP25	15	2DB25

### Details of beams

**Figure 2:**
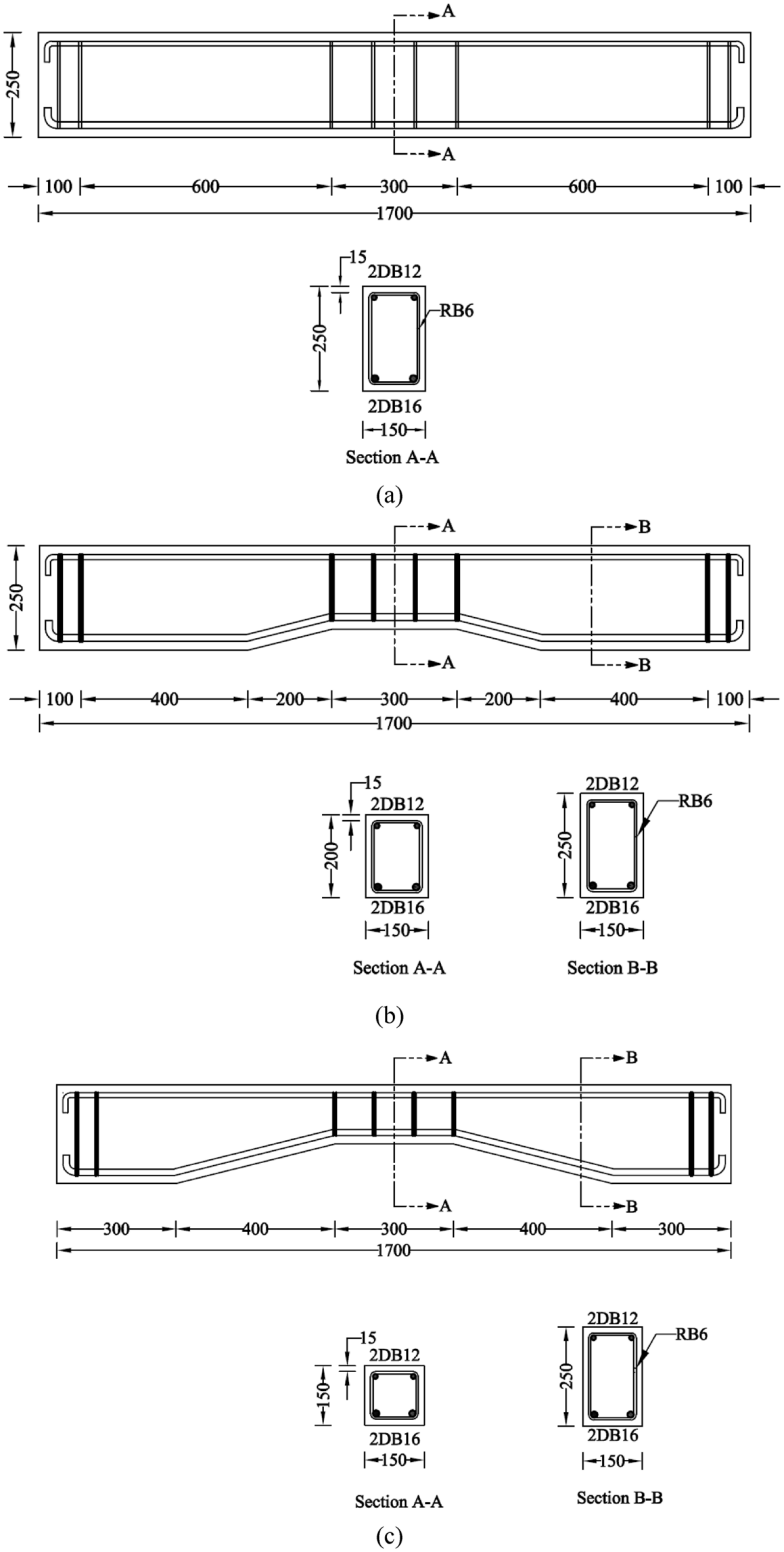

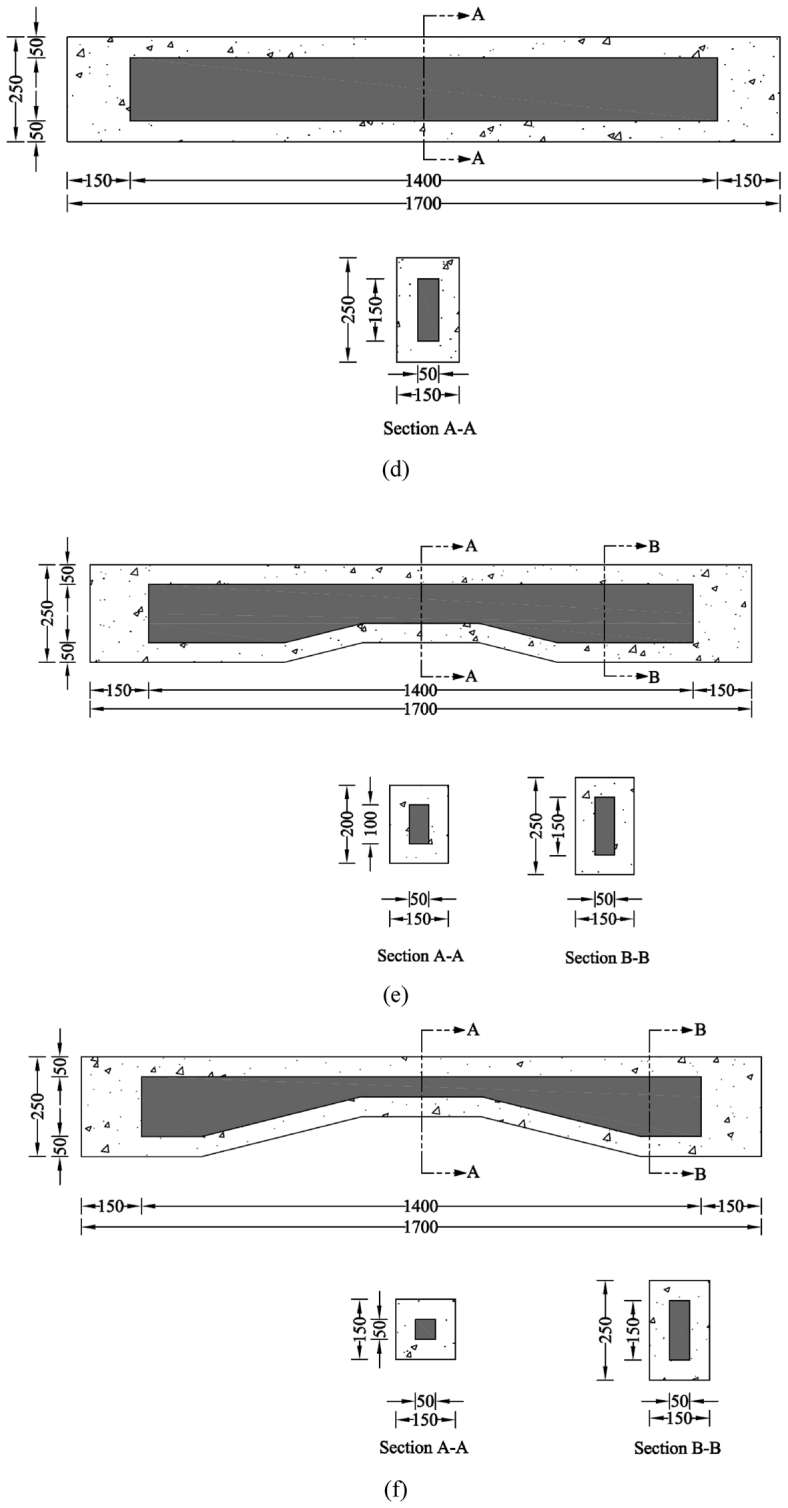
Structural details of tested beams (**a**) P-S-01, (**b**) NP-S-02, (**c**) NP-S-03, (**d**) P-H-04, (**e**) NP-H-05, (**f**) NP-H-06.

Each beam had a total length of 1700 mm, a height of 250 mm, and a width of 150 mm. The structural details of all beams are shown in Fig. 2. All beams were longitudinally reinforced with two 16 mm-deformed beams on the tension side and two 12 mm-deformed bars on the compression side. Four stirrups of 6 mm-round bars were provided within the midspan at a spacing of 100 mm. The longitudinal reinforcement was provided with $90^\circ$ hooks near beam ends. Specimen P-S-01 had a prismatic solid section. Specimen NP-S-02 had a prismatic solid section of 500 mm at its ends, followed by a tapered solid section of 200 mm, and a prismatic solid section again within the midspan for 300 mm. Specimen NP-S-03 had an increased tapered length of 400 mm. As a result, the depth of the section at midspan was 150 mm in contrast to the depth of 200 mm in NP-S-02. Specimens P-H-04, NP-H-05, and NP-H-06 were structurally similar to specimens P-S-01, NP-S-02, and Beam-03, respectively, with the difference of hollow section only. A rectangular opening of size 50 mm $\times$ 150 mm was provided concentrically in Specimen P-H-04. The opening size in Specimen NP-H-05 was reduced to 50 mm $\times$ 100 mm at the midspan, whereas the same reduction in Specimen NP-H-06 was to the size of 50 mm $\times$ 50 mm at its midspan.

### Material properties

The mechanical properties of steel reinforcement were estimated by following the recommendations of ASTM E8/E8M-21^42^. Standard tensile tests by using three samples were carried out to determine the stress–strain curves of all steel bars. Table 3 summarizes the estimated properties of steel reinforcement. Concrete cylinders were fabricated by pouring concrete in three equal layers with each layer subjected to a uniform compaction with the help of tamping rod. Similarly, beams the compaction of beams was performed by using a mechanical vibrator. Curing was performed in laboratory condition for a period of 28 days. The target concrete strength for all beams was 15 MPa, whereas the actual concrete strength estimated by the recommendations of ASTM C39^43^ was 15.20 MPa. Similar to the tension steel bars, low-strength concrete was considered in the experimental investigations to accommodate the laboratory test equipment such as reaction frame, load cell, and hydraulic jack.

**Table 3:** Mechanical properties of steel reinforcement.

Bar type	Yield strength (MPa)	Tensile strength (MPa)
RB6	372.70	494.70
DB12	512.20	650.10
DB16	502.74	646.38

### Construction of RC beams

**Figure 3:**
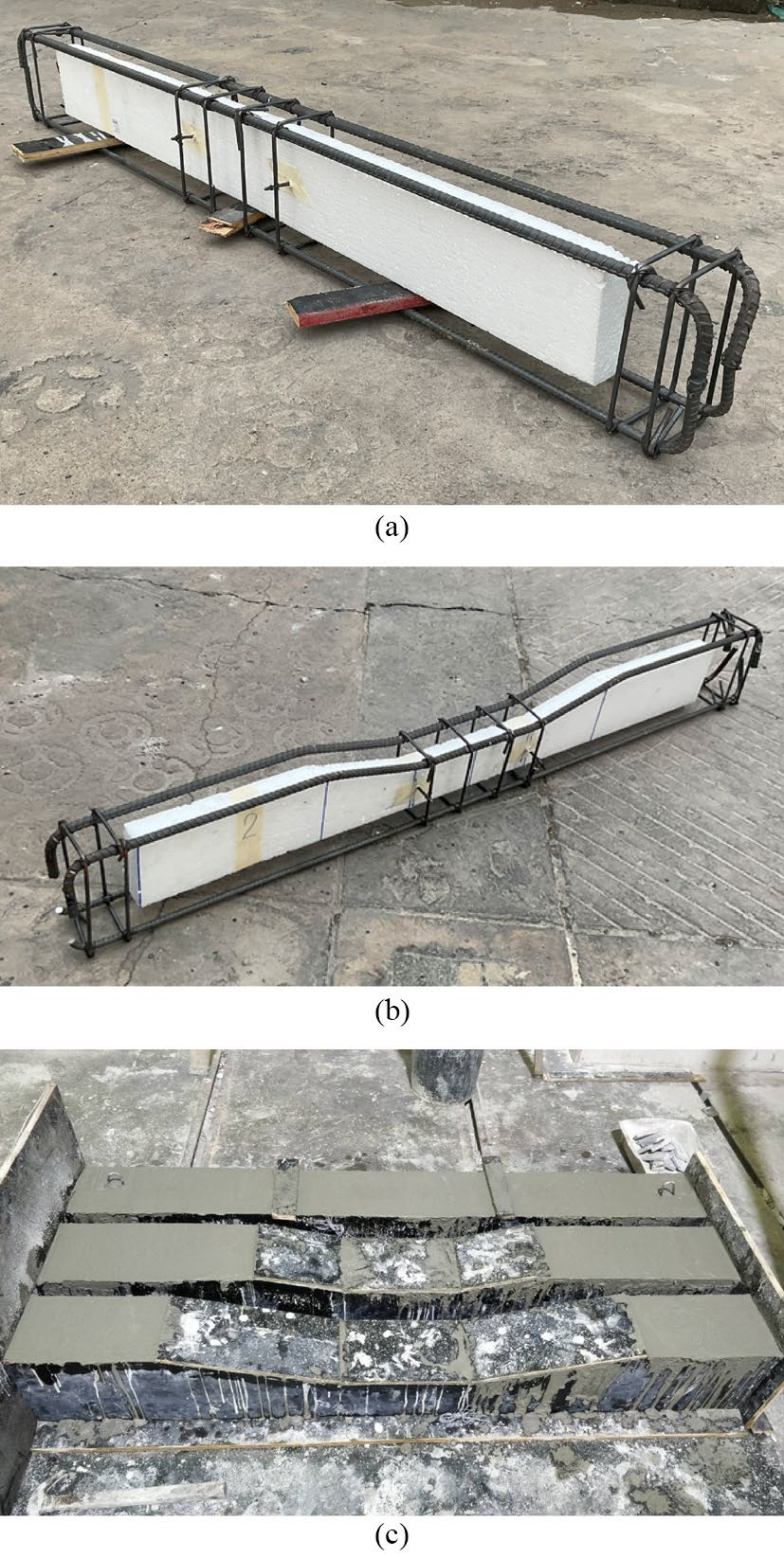
Construction of RC beams (**a**) typical Styrofoam in prismatic beam, (**b**) typical styrofoam in steel bars, (**c**) construction of RC beams.

The construction of the hollow prismatic and non-prismatic sections was carried out with the help of Styrofoam, as shown in Fig. 3. A piece of Styrofoam was cut into the required dimensions and placed inside the steel cage. Wooden spacers were used in combination with transverse steel rods at the midspan to maintain the position of Styrofoam. Further, plywood sheets were used as formwork to cast the concrete, as shown in Fig. 3.

### Test setup and instrumentation

**Figure 4:**
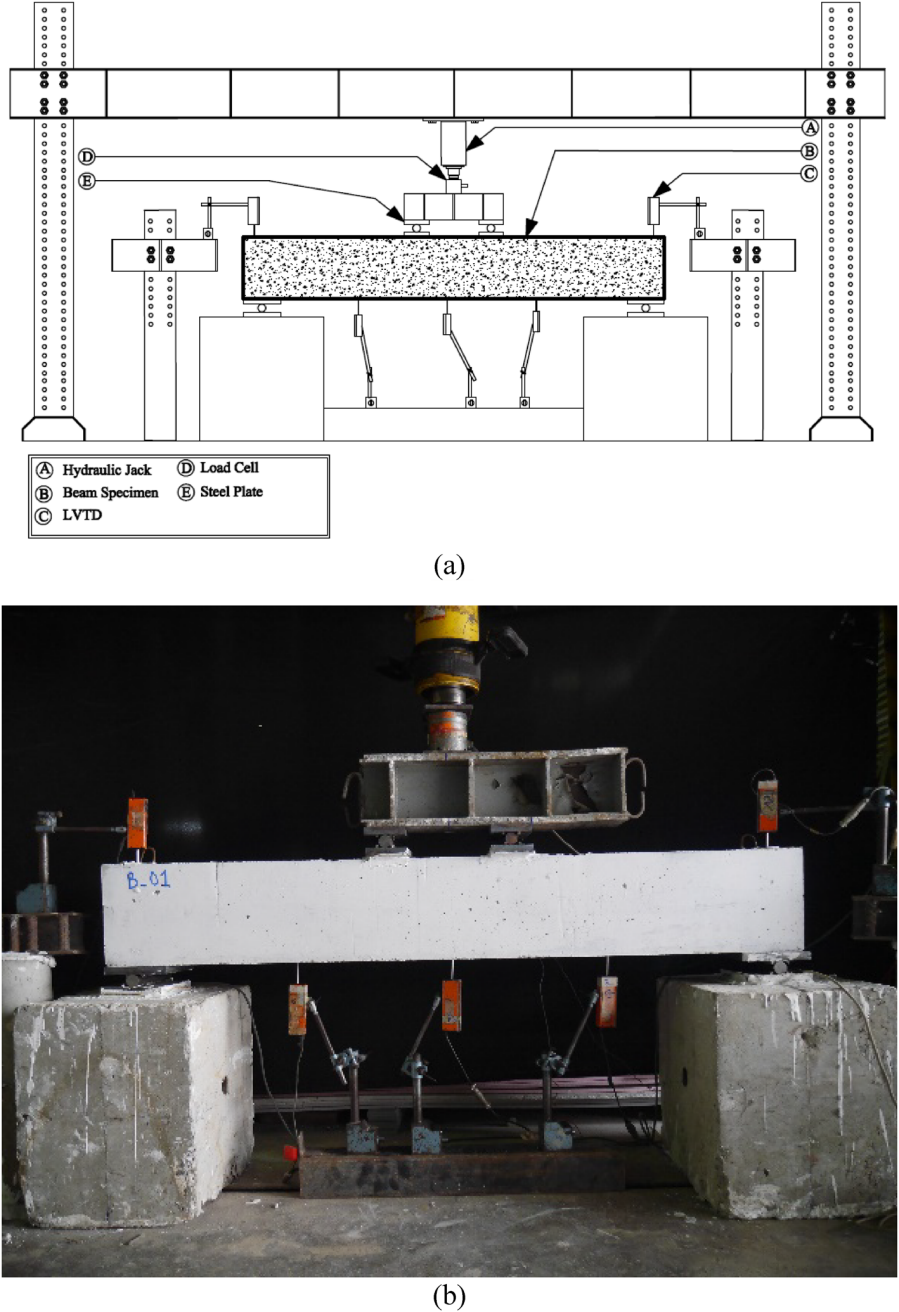
Test setup (**a**) schematics, and (**b**) actual setup.

Each beam was subjected to a monotonic four-point bending, as shown in Fig. 4. The load was applied by using a hydraulic jack, and the intensity of the applied load at each time instant was monitored by using a load cell. The vertical deflection of the beam was measured by using three displacement transducers. Two additional displacement transducers were attached to the ends to measure any unwanted support uplift. One strain gauge was mounted at the top longitudinal bar at midspan, whereas another strain gauge was mounted to the bottom longitudinal bar.

## Experimental results

### Failure modes

**Figure 5:**
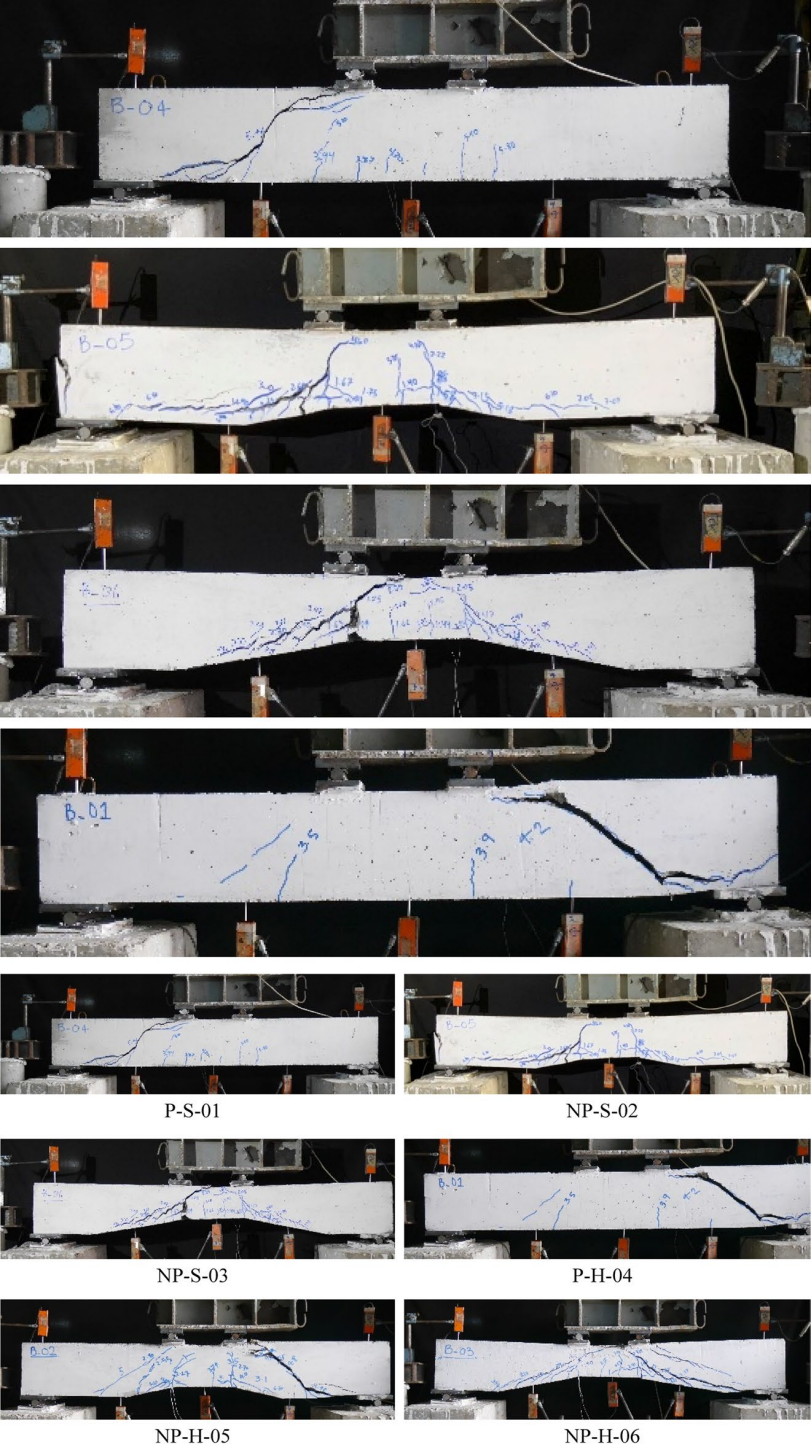
Ultimate failure modes of beams.

The failure of all beams is shown in Fig. 5. The failure of Specimen P-S-01 was accompanied by flexural cracks near its soffit. At a load of 30 kN, flexural cracks started to appear and propagated toward the neutral axis. The increase in load resulted in the appearance of additional flexural cracks. At a load of around 62 kN, an inclined crack appeared staring from the left loading plate towards the left support. Consequently, the beam lost its capacity in a brittle manner. Specimen P-S-01 was unable to demonstrate any significant vertical deformation. The Specimen NP-S-02 demonstrated several flexural cracks during the initial loading stage. The first flexural crack appeared near a load of 14 kN. This beam was able to exhibit vertical deflection at the initial loading stage. At a load of 70 kN, a shear crack was formed between the left loading plate and the left support. The shear crack was joined by already present flexural cracks near its vicinity. The drop in load capacity and hence the failure of Specimen NP-S-02 was less brittle as compared to that of Specimen P-S-01. The failure of Specimen NP-S-03 was the least brittle among the beams with solid sections. It can be seen in Fig. 5 that Specimen NP-S-03 did not demonstrate a shear crack. The inclined shear crack that originated from underneath the left loading plate was unable to propagate to the left support. As a result, a ductile response was exhibited by Specimen NP-S-03.

The failure of Specimen P-H-04 was identical to that of Specimen P-S-01. A wide shear crack was observed near the load of 42 kN. Following that, the load capacity dropped suddenly. Specimen NP-H-05 exhibited an identical response to that of Specimen NP-S-02. Several flexural cracks appeared within the tension zone before the formation of the final shear crack at a load of 65 kN. Finally, the failure of Specimen NP-H-06 was identical to that of Specimen NP-S-03. As shown in Fig. 5, Specimen NP-H-06 exhibited several inclined cracks, but the formation of these cracks was slow and did not result in a sudden drop in its load capacity.

### Ultimate load and deflection

**Figure 6:**
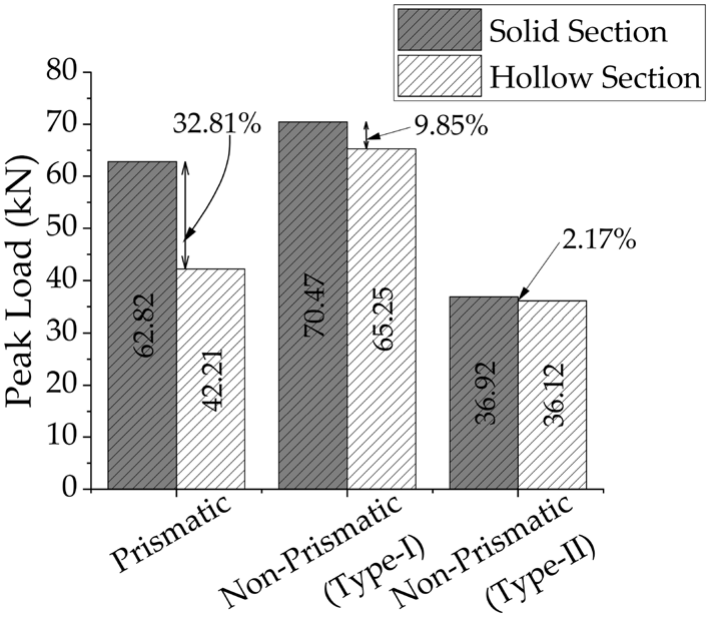
Comparison of peak loads of beams.

A summary of the ultimate load and deflection is presented in Table 4. Specimen P-S-01 was able to withstand a maximum load of 62.82 kN, whereas the corresponding deflection was 2.51 mm. Specimen NP-S-02 demonstrated a higher peak load of 70.47 kN than that Specimen P-S-01. In addition, the deflection at peak load was significantly higher as well. The length of the tapered section was further increased in Specimen NP-S-03. This resulted in a further increase in deflection at ultimate load, whereas the peak sustained load least among specimens with solid section. It can be observed that by increasing the tapered length along the beam, the flexibility of the beam increased. A similar trend was also observed in specimens with hollow sections. A comparison of the peak loads of specimens P-S-01 and NP-S-02 suggests that a hollow section beam demonstrated a 32.81% lower capacity than that of the same beam but with a solid section (see Fig. 6). On the contrary, this reduction in peak capacity was insignificant in the case of the non-prismatic section. For instance, the difference in the peak sustained loads of specimens NP-S-02 and NP-H-05 was 9.85% (see Fig. 6). The same difference was reduced to 2.17% in the case of specimens NP-S-03 and NP-H-06 (see Fig. 6). Hence, it can be concluded that beams with hollow non-prismatic sections can have the same capacity as those of the same beams but with solid sections. However, the beams with prismatic sections can have significantly different capacities depending upon the presence of longitudinal openings.

**Table 4:** Summary of ultimate load, deflection, and dissipated energy.

Type of beams	Peak load (kN)	Deflection again peak load (mm)	Accumulated dissipated energy (kN-mm)
P-S-01	62.82	2.51	319.45
NP-S-02	70.47	16.26	647.46
NP-S-03	36.92	19.76	1041.87
P-H-04	42.21	1.89	330.23
NP-H-05	65.25	9.97	1397.78
NP-H-06	36.12	15.76	975.29

### Load–deflection curves

**Figure 7:**
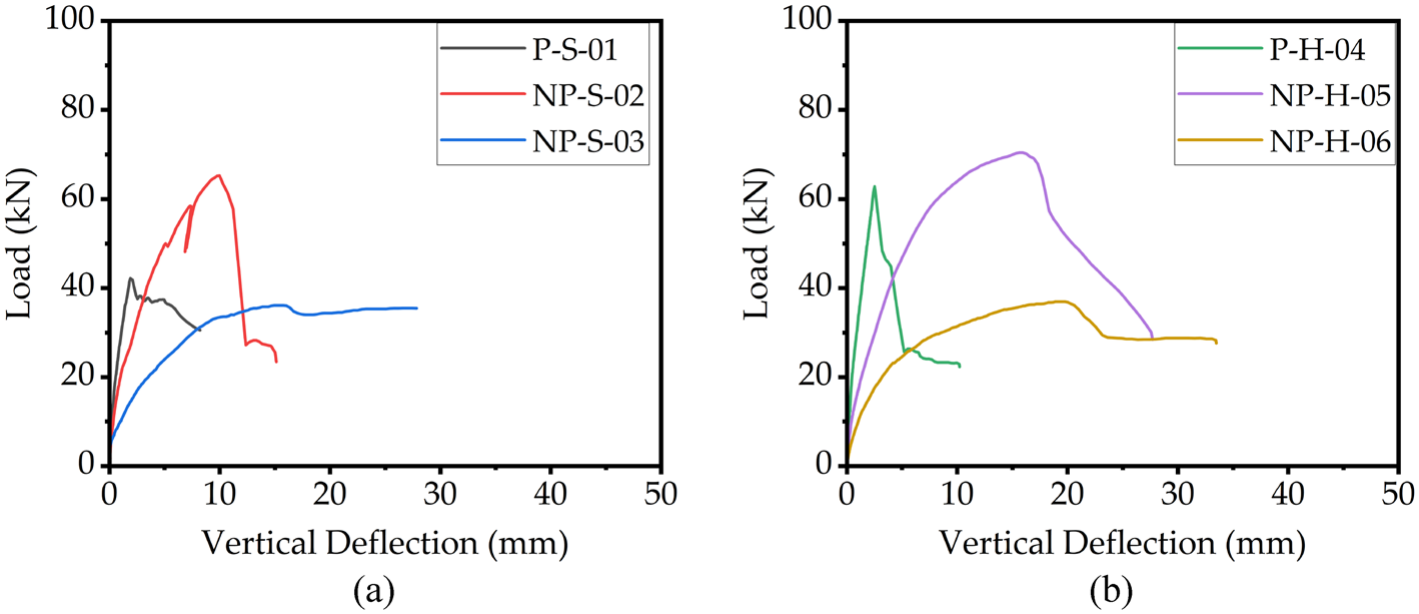
Experimental load–deflection curves (**a**) solid cross-section beams and (**b**) hollow cross-section beams.

**Figure 8:**
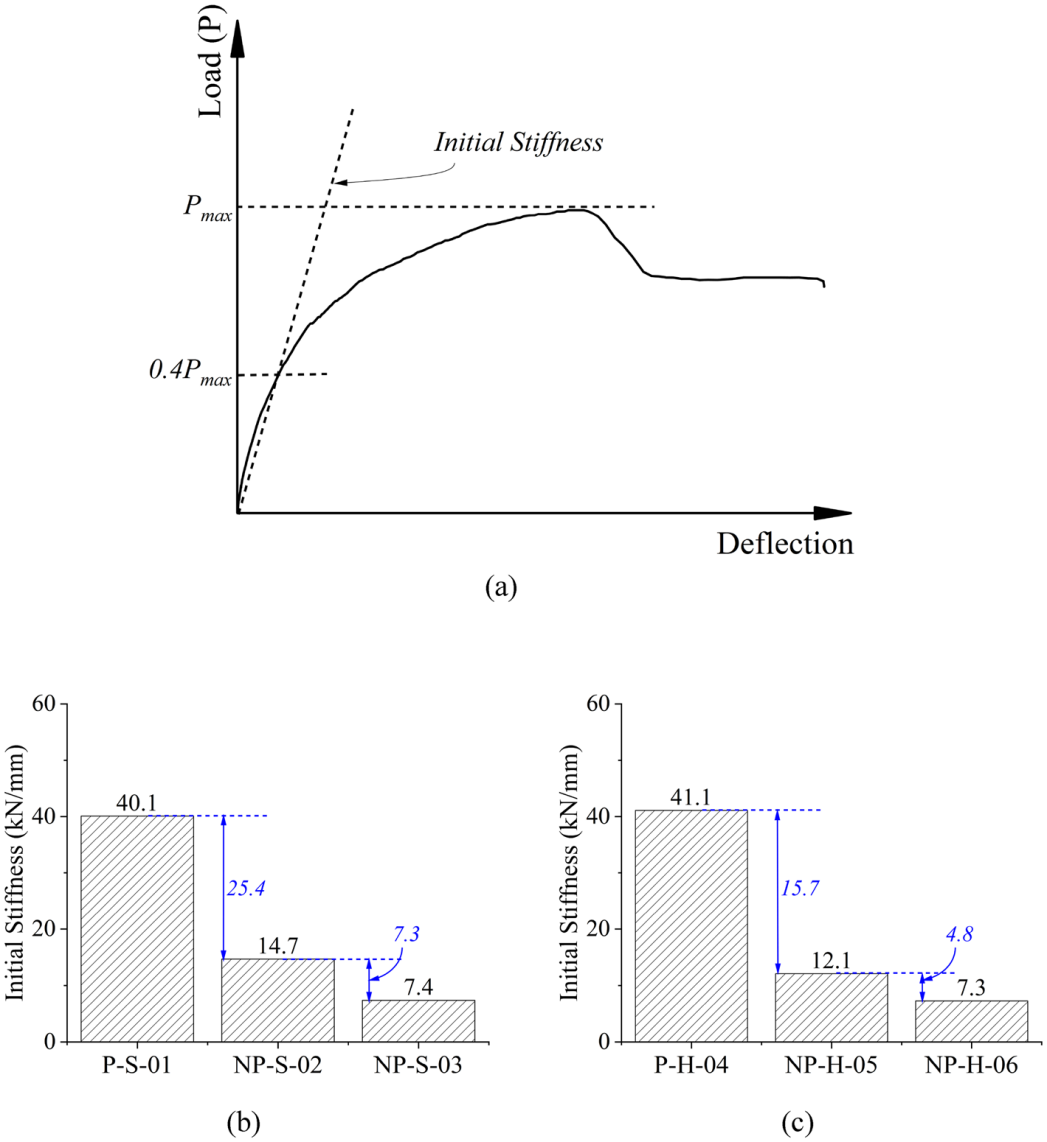
(**a**) Definition of initial stiffness, (**b**) comparison of initial stiffness of solid section beams, and (**c**) comparison of initial stiffness of hollow section beams.

The load–deflection curves of all beams are shown in Fig. 7. It is to be noted that the initial stiffness of load–deflection curves was estimated by joining a line from origin to the point that corresponded to the 40% peak capacity^44^, as shown in Fig. 8a, whereas the comparison of computed initial stiffness is shown in Fig. 8b,c. Several important conclusions can be drawn: (1) the initial stiffness was maximum in the case of prismatic sections, i.e., for specimens P-S-01 and P-H-04, (2) the inclusion of non-prismatic section reduced the initial stiffness. This reduction was proportional to the length of the non-prismatic section along the beam. For instance, NP-S-02 had lower initial stiffness than that P-S-01, whereas NP-S-03 had the least initial stiffness among specimens P-S-01, NP-S-02, and NP-S-03, (3) ductility of beams increased as the length of the non-prismatic section along the beam increased. This suggests that a beam with a non-prismatic section is less prone to shear failure than a beam with a prismatic section. However, this may be achieved at the expense of a reduced peak load capacity, (4) the peak load was higher for the solid beam than the peak load of the hollow beam. This observation was prominent in prismatic section beams.

### Strain of steel bars

**Figure 9:**
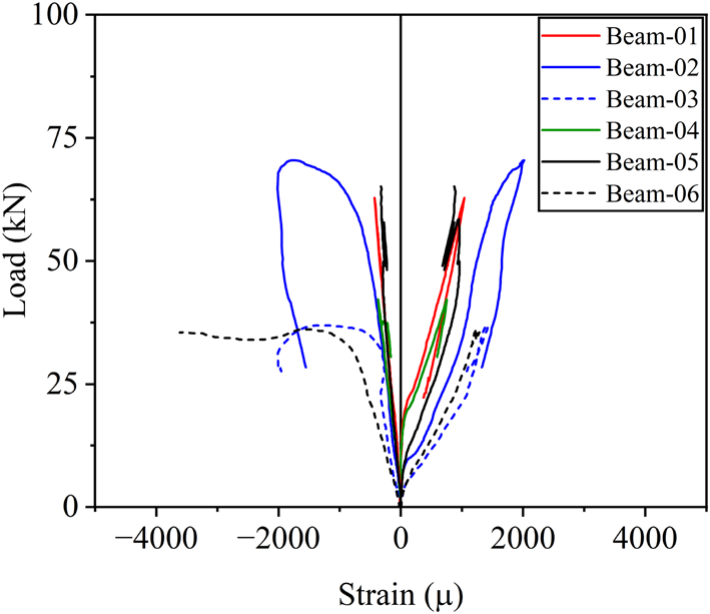
Comparison of longitudinal bar strains on tension and compression sides.

The strain gauge measurements along the top and bottom longitudinal bars are shown in Fig. 9. The maximum positive strain in specimens P-S-01, NP-S-02, and NP-S-03 were 1034 microns, 2016 microns, and 1430 microns, respectively. This shows that the inclusion of a tapered section allowed the beam to achieve a better structural response. The maximum positive strain in specimens P-H-04, NP-H-05, and NP-H-06 were 752 microns, 960 microns, and 1287 microns, respectively. The inclusion of openings also reduced the maximum positive strain along longitudinal bars. For instance, the maximum positive strain along longitudinal bars in specimens P-S-01 and P-H-04 were 1034 microns and 752 microns, respectively. The maximum negative strain in specimens P-S-01, NP-S-02, and NP-S-03 were -432 microns, -2020 microns, and -2016 microns, respectively. Similarly, the maximum negative strain in specimens P-H-04, NP-H-05, and NP-H-06 were − 374, − 326, and − 3620 microns, respectively. Clearly, a tapered beam was able to withstand higher steel strains both on the negative and positive sides. However, solid section beams were able to achieve higher negative strains than those of hollow section beams.

### Accumulated dissipated energy

The total energy dissipated by each specimen was computed by integrating the areas under load–deflection curves. The last column of Table 4 presents the computed accumulated dissipated energy of all specimens. For beams with solid sections, the least dissipated energy belonged to Specimen P-S-01, with a value of 319.45 kN-mm. The inclusion of a tapered section in Specimen NP-S-02 increased this value to 647.46 kN-mm. The length of the tapered section was further increased in Specimen NP-S-03. Correspondingly, the accumulated dissipated energy increased to 1041.87 kN-mm. For hollow section beams, the least dissipated energy also belonged to Specimen P-H-04, i.e., with a prismatic section. The inclusion of tapered sections in Specimens NP-H-05 and NP-H-06 increased the dissipated energy to 1397.78 kN-mm and 975.29 kN-mm, respectively. Thus, in general, beams with non-prismatic sections were able to demonstrate higher energy dissipation capacities than beams with prismatic sections. Further, the energy dissipation capacities of beams with non-prismatic hollow sections were higher than beams with non-prismatic solid sections.

## Finite element modeling

**Figure 10:**
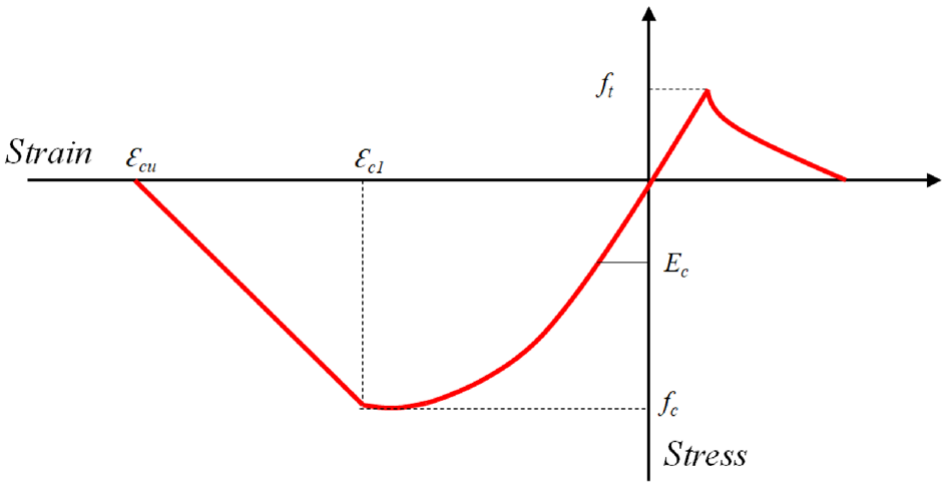
Stress–strain relation adopted for concrete (${f}_{c}{\prime}=15 MPa, {E}_{c}=18.2 GPa, {\epsilon }_{c1}=0.002, {f}_{t}=1.5 MPa, {\epsilon }_{c2}=0.0048$).

The nonlinear finite element modeling was performed by using *ATENA*^45^. In the first step, FEM was performed and validated against experimental results of the present study, similar to existing studies^46,47^. Then, FEM was extended to study the effects of concrete strength glass fiber-reinforced bars on the structural performance of beams. The concept of smeared cracking is utilized in ATENA for nonlinear modeling. The 8-node solid element *CC3DNonLinCementitious2* was used to model concrete. The global mesh size was kept at 0.015 m. The uniaxial stress–strain relation shown in Fig. 10 was used for concrete.

**Figure 11:**
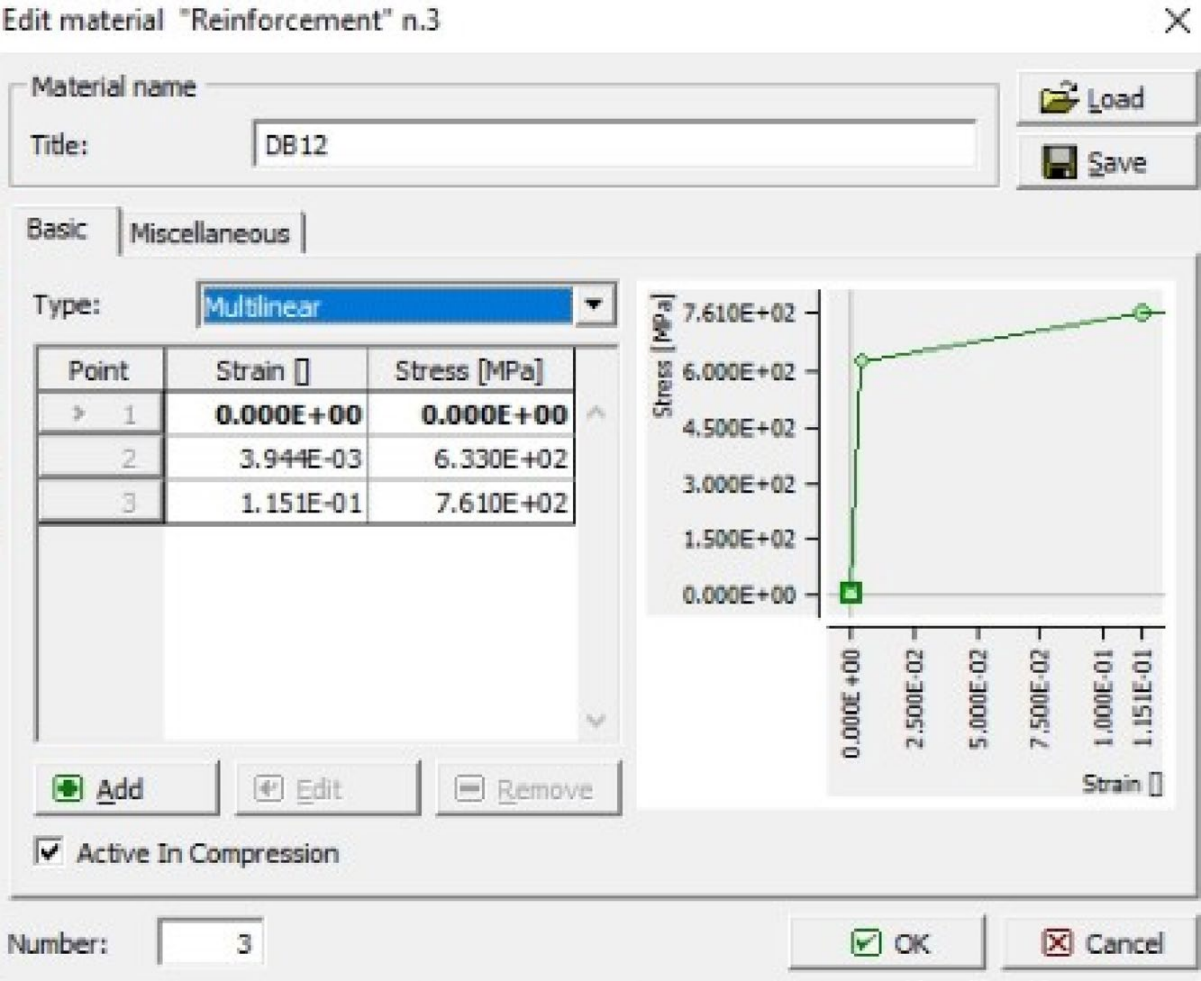
Definition of multilinear curve to model stress–strain behavior of steel bars.

**Figure 12:**
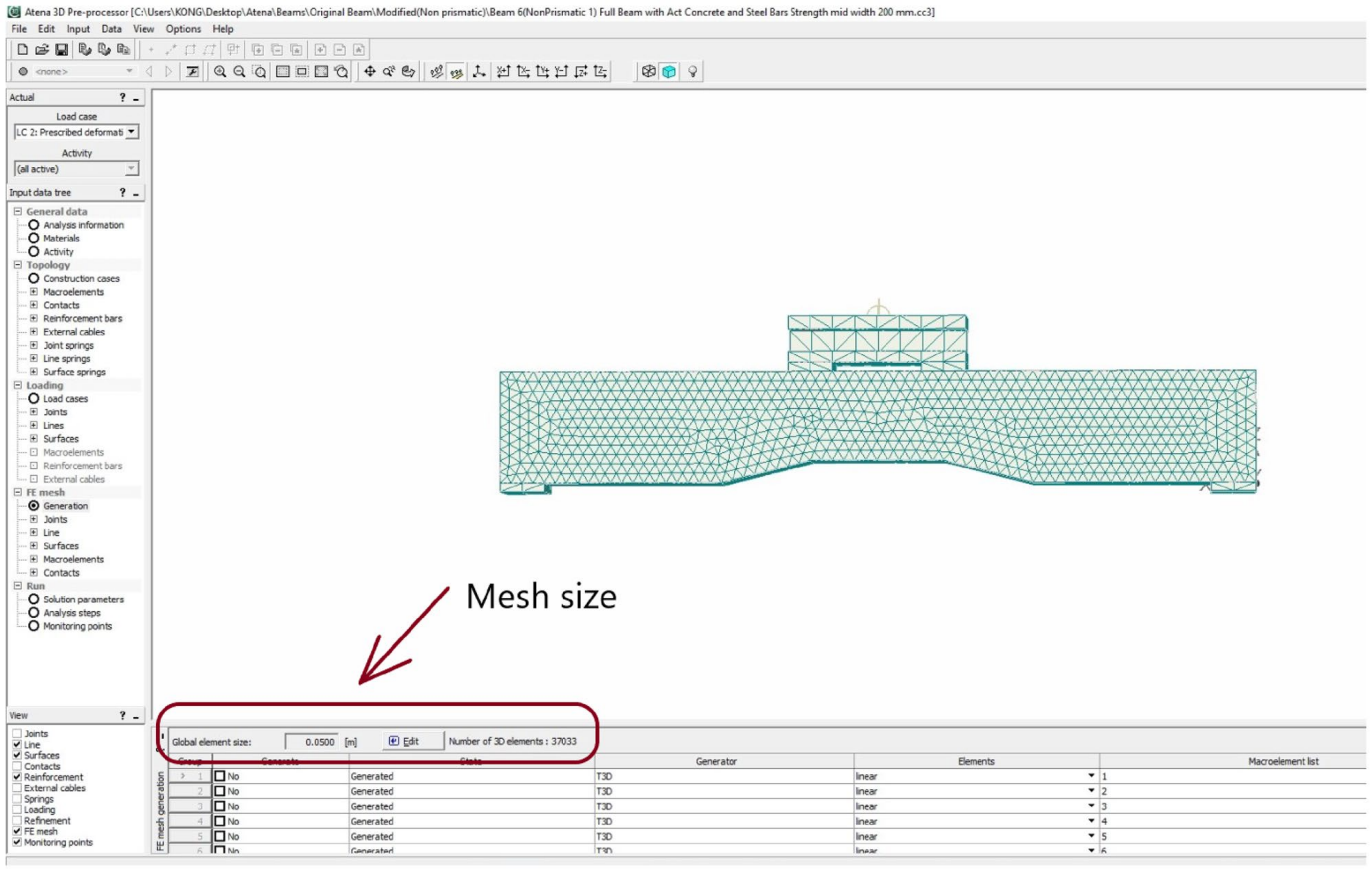
Typical display of mesh size in ATENA.

**Figure 13:**
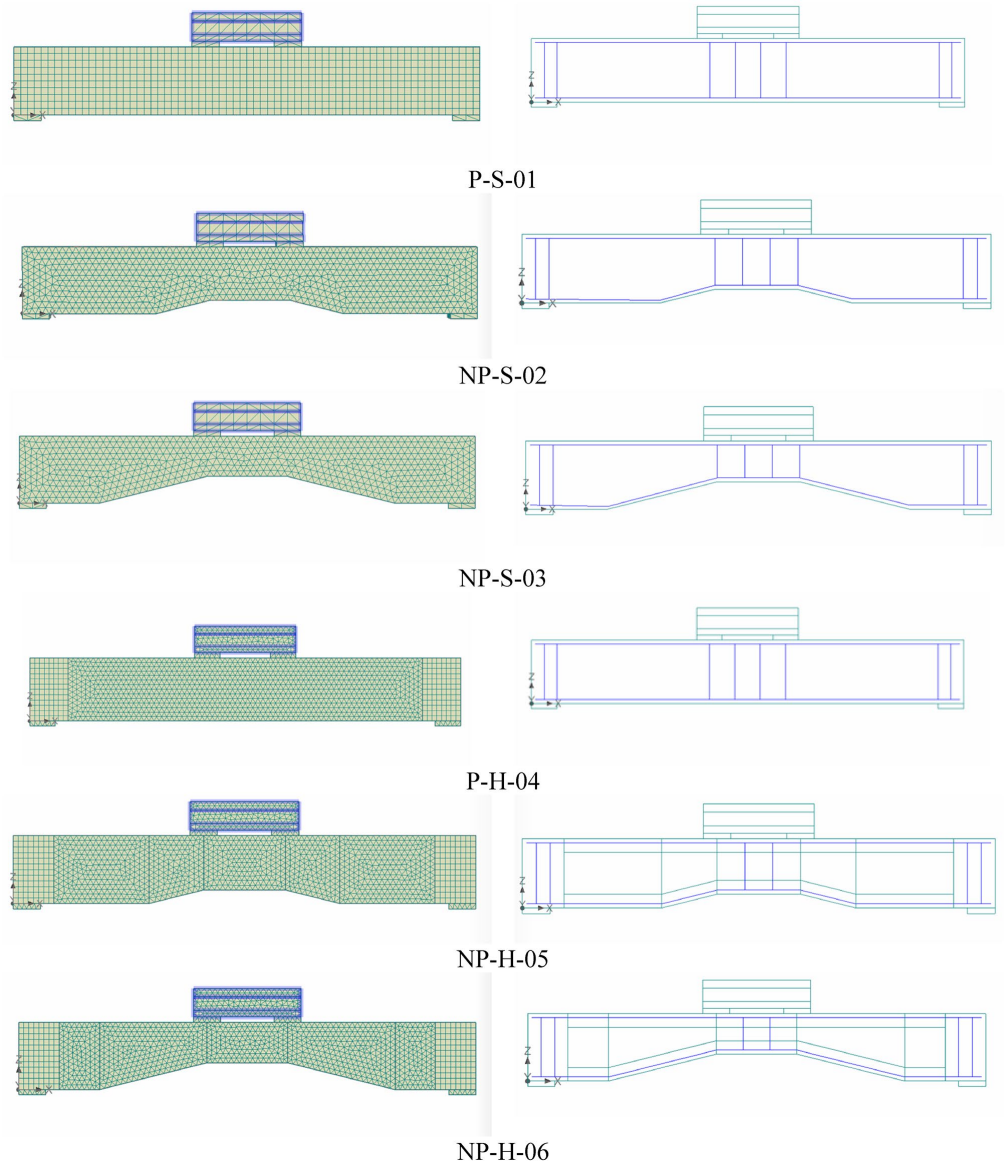
Mesh and reinforcement details of FEM.

Linear elastic behavior was assumed for concrete before cracking in tension, whereas a fictitious model based on crack-opening law and fracture energy was utilized for post-cracking in tension. The ascending compressive branch was modeled by using the recommendations of CEB-FIP^48^. The steel reinforcing bars were modeled by using a multilinear curve to allow for modeling various stages along the constitutive stress–strain relation of steel bars. The multilinear curve was assigned to built-in truss materials in ATENA. The definition of a typical multilinear curve for steel bars in ATENA is shown in Fig. 11. The slip between steel bars and concrete was neglected by assuming a perfect bond. In this study, parametric analysis was conducted to select appropriate mesh size to accommodate the accuracy of results, analysis time and storage capacity of the computer. Based on the parametric analysis, global mesh size was selected as 0.05 m was selected in the ATENA (Fig. 12). The meshing and reinforcement details are shown in Fig. 13. In the past^49,50^, linear stress versus strain responses were observed for GFRP rebars, therefore in this study, the GFRP rebars were modelled as linear curve using built-in truss element *CCReinforcement*.

**Figure 14:**
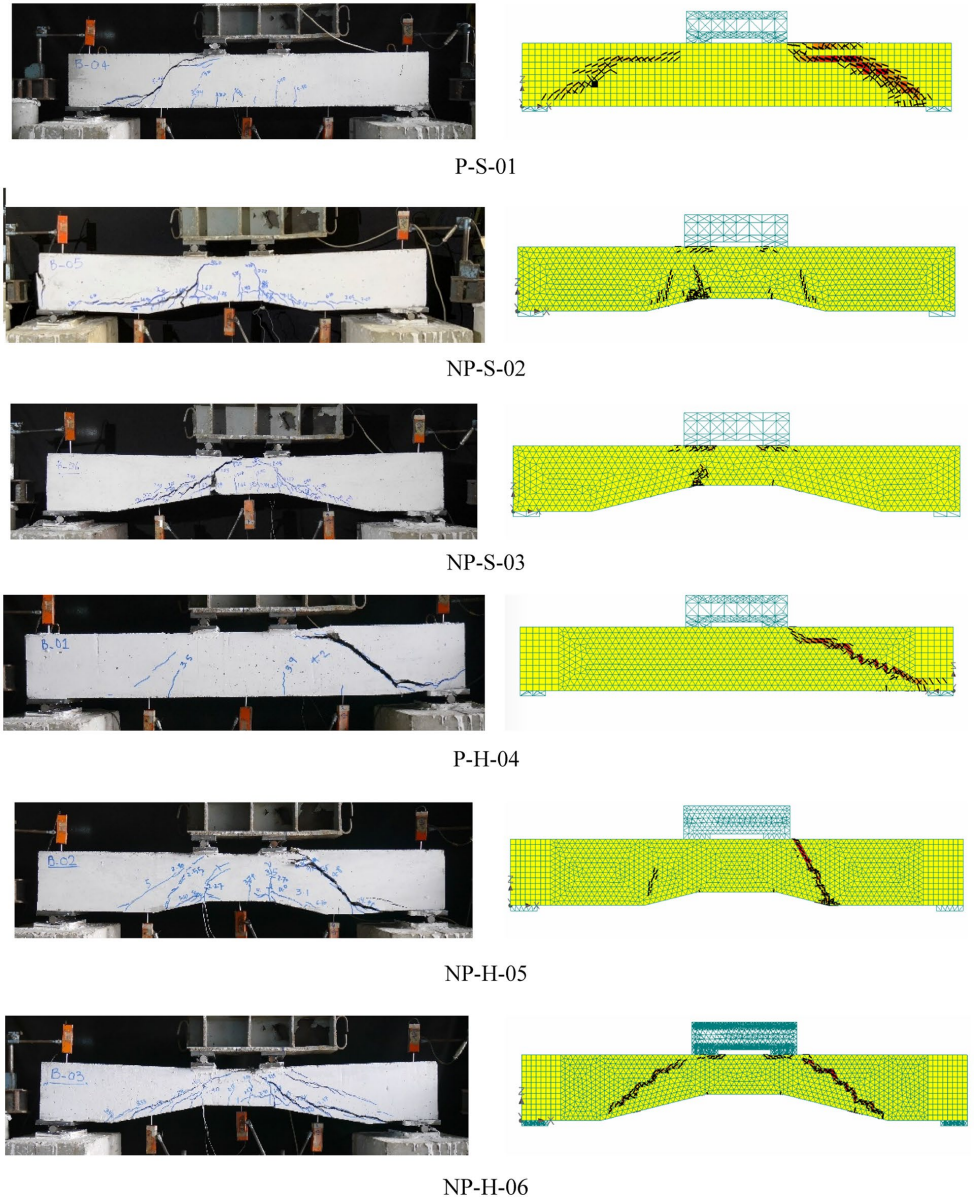
Comparison of experimental and analytical crack patterns at the ultimate stage.

The comparison of crack patterns at the ultimate failure of beams is shown in Fig. 14. In general, the adopted modeling strategy resulted in close agreement with experimental crack patterns for all beams. Inclined shear cracks between loading points and support plates were observed in solid and hollow prismatic beams. The cracks observed in non-prismatic beams were lesser than those in prismatic beams. This was confirmed by their corresponding experimental results. Table 5 summarizes the comparison of experimental and analytical ultimate loads of all beams. The ultimate loads predicted by the nonlinear modeling were generally higher than their corresponding experimental values.

**Table 5:** Comparison of experimental and FEM predicted ultimate loads.

Beam ID	Ultimate failure load (kN)		Difference (%)
	Experiment	FEM	
P-S-01	62.82	70.02	11.46
NP-S-02	70.47	74.83	6.19
NP-S-03	36.92	52.86	43.17
P-H-04	42.21	51.00	20.82
NP-H-05	65.25	57.42	-12.00
NP-H-06	36.12	44.64	23.58

### Parametric finite element modeling

Although many studies have reported the behavior of non-prismatic RC beams, however, the effect of the strength of concrete was not studied for different types of non-prismatic RC beams with solid and hollow sections. Further, the effect of the longitudinal reinforcement ratio was also not considered in the previous studies. Therefore, in this section, parametric finite element modeling was performed to study the effect of the strength of concrete and longitudinal reinforcement ratio on the ultimate load-carrying capacity and cracking behavior of prismatic and non-prismatic RC beams with solid and hollow sections. The developed finite element models were further extended, and a total number of 12 RC beams were modeled in ATENA. The details of parametric RC beams are shown in Table 2. The results of parametric finite element analysis are discussed in the following sections.

#### Effect of concrete strength

**Figure 15:**
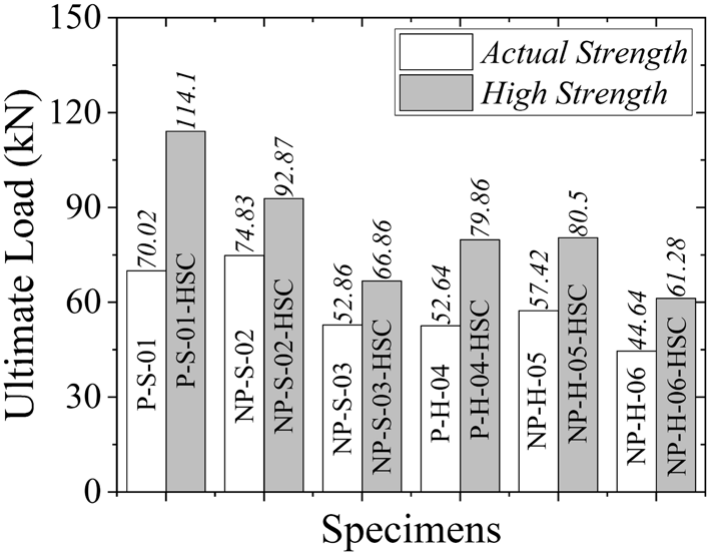
Comparison of ultimate loads predicted by FEM for actual and high concrete strength.

**Figure 16:**
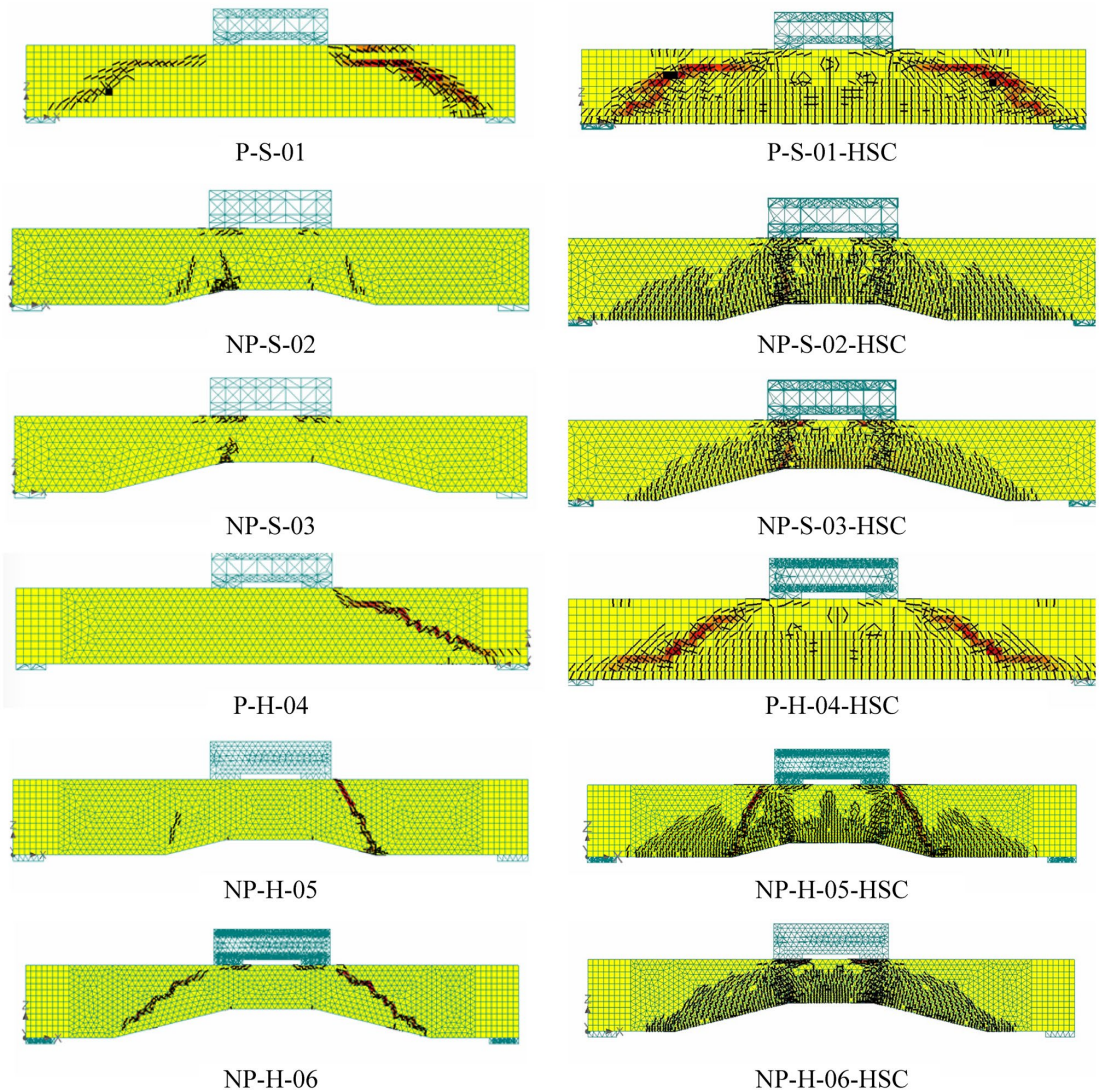
Comparison of FEM cracking results (left column) actual concrete strength and (right column) concrete strength doubled.

Figure 15 presents the comparison of ultimate loads predicted by FEM for actual and high-strength concrete RC beams. It is evident that ultimate loads increased as the concrete strength increased. Figure 16 presents the comparison of FEM results with actual concrete strength (left column) and FEM results with high concrete strength (right column). By increasing the concrete strength, the moment capacity of concrete was expected to increase. Consequently, more flexural cracks within the tension zone were expected. This was confirmed by FEM results (see right column of Fig. 16) with increased intensity of flexural cracks. However, the main crack orientation remained the same, indicating that failure patterns would not alter by increasing the concrete strength. In general, wider cracks were observed for high-strength concrete.

#### Effect of reinforcement ratio

**Figure 17:**
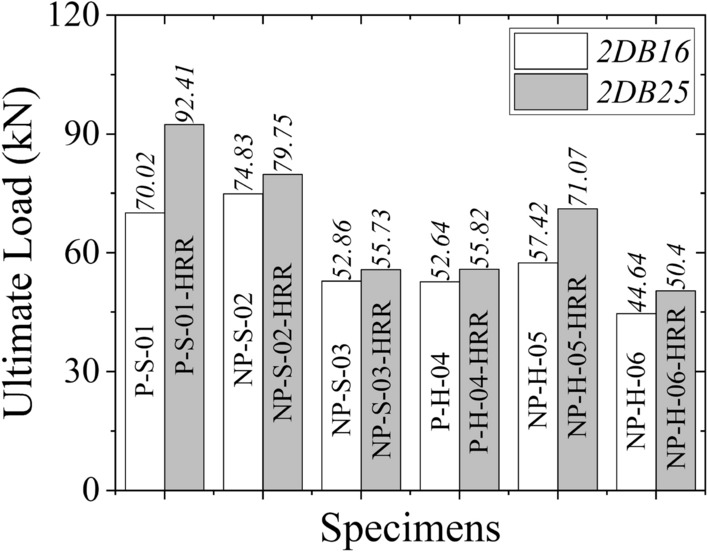
Comparison of ultimate loads predicted by FEM for actual and high longitudinal reinforcement ratio.

**Figure 18:**
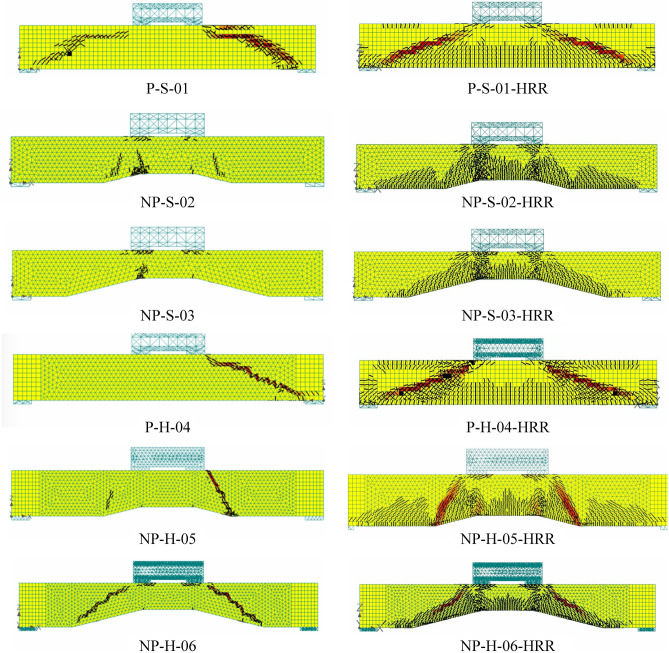
Comparison of FEM cracking results (left column) actual longitudinal bars and (right column) 25 mm longitudinal bars.

The ultimate loads increased as the longitudinal reinforcement ratio increased (see Fig. 17). The increase in longitudinal reinforcement ratio had a similar effect on crack patterns and their width as that of the concrete strength. Wider and more intense cracks were observed by increasing the longitudinal reinforcement ratio. The results of the second parametric study are shown in Fig. 18. The beams were again expected to sustain higher moment capacities as a result of an increased cross-sectional area of steel bars. This was reflected in FEM results in terms of more flexural cracks (see right column of Fig. 18). More importantly, the failure crack patterns were not changed. Thus, it can be established that the failure patterns observed in the present study will not change if the longitudinal reinforcement ratio is increased. Further, the failure patterns were found to be independent of the size of longitudinal bars.

#### Effect of GFRP rebars

**Figure 19:**
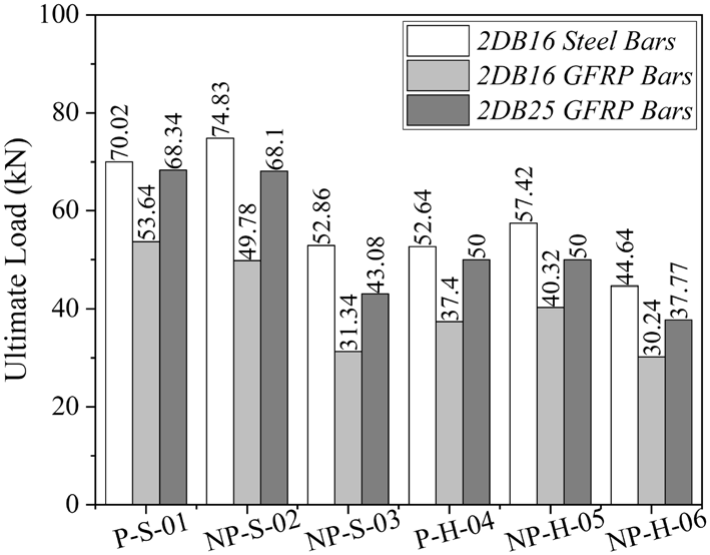
Comparison of ultimate loads predicted by FEM for steel bars and GFRP bars.

**Figure 20:**
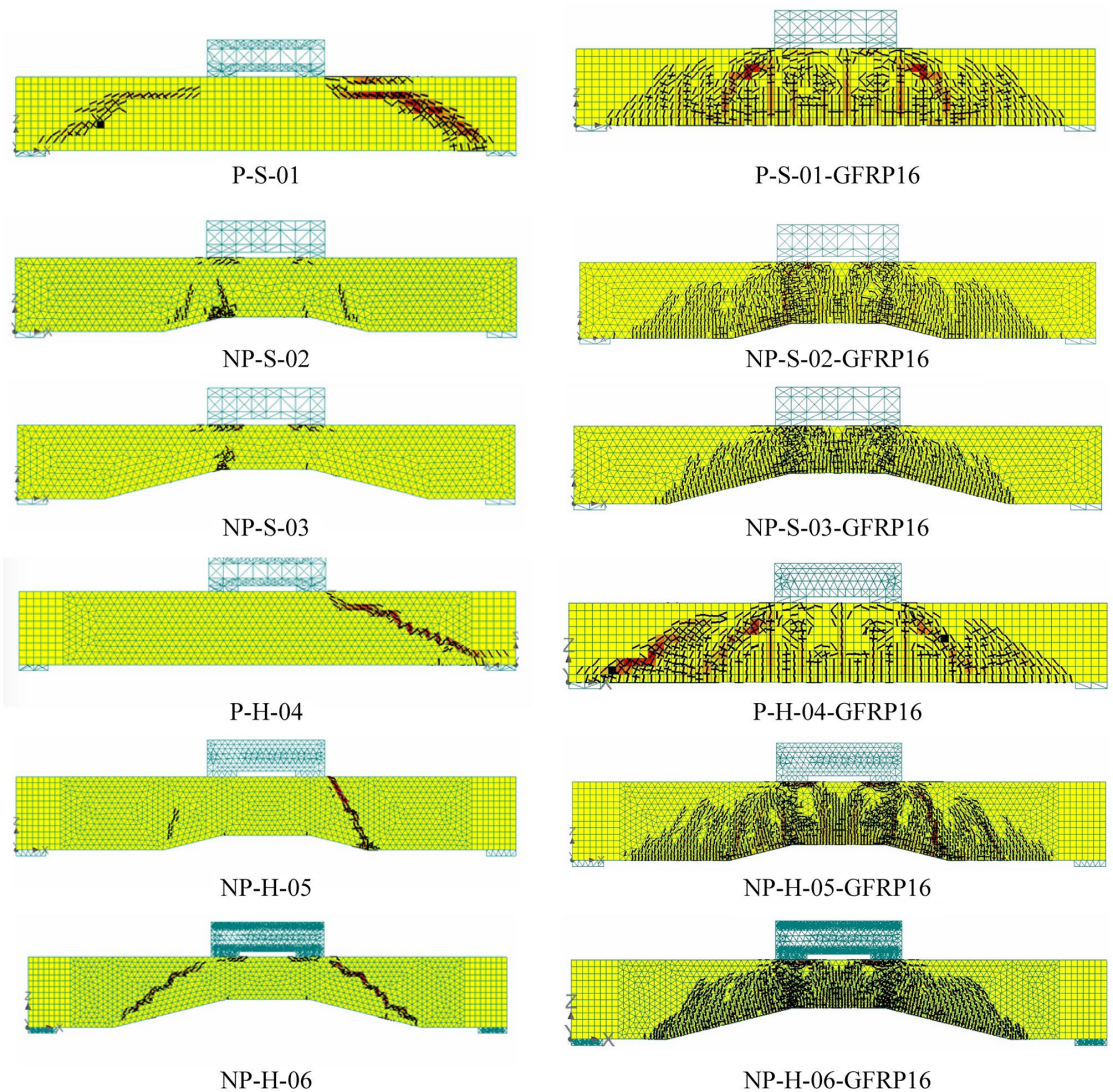
Comparison of FEM cracking results (left column) actual beams and (right column) beams with 16 mm GFRP bars.

**Figure 21:**
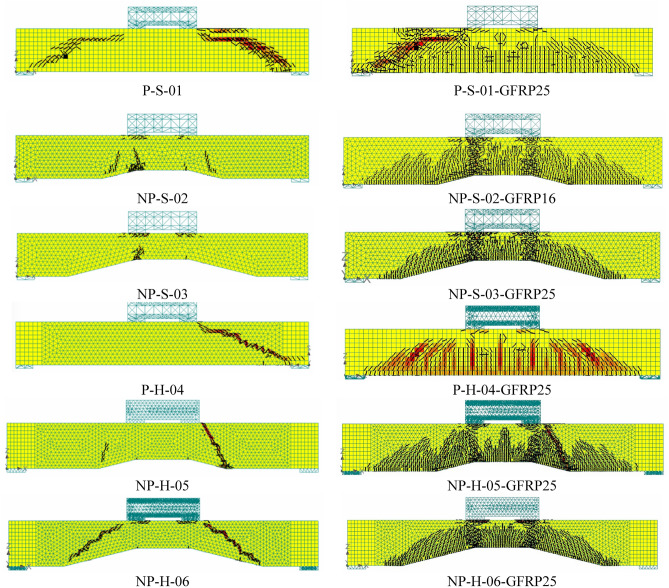
Comparison of FEM cracking results (left column) actual beams and (right column) beams with 25 mm GFRP bars.

A comparison of the ultimate loads predicted by FEM for beams with steel bars and different sizes of GFRP bars is shown in Fig. 19. By comparing the response beams with steel and GFRP bars of the same diameter, a close agreement between the ultimate capacities was observed, with a difference of only 23%. This difference was further reduced when the diameter of GFRP bars was increased to 25 mm. The comparison of cracking patterns predicted by FEM for steel and GFRP bars of 16 mm diameter is shown in Fig. 20. It is recalled that GFRP bars were used for both the longitudinal and shear reinforcement. The cracking patterns for beams with steel and GFRP bars were comparable, with GFRP bars showing a slightly larger number of diagonal cracks. Thus, it can be inferred that GFRP bars tend to resist shear forces in a similar way as that of steel bars. The number and extent of flexural cracks in GFRP-strengthened beams were similar to that of steel-reinforced beams. A similar behavior was observed in the case of beams longitudinally reinforced with GFRP bars of 25 mm diameter (see Fig. 21). The failure of beam is initiated by the yielding of steel bars in steel reinforced beams, whereas the fracture of GFRP bars control the failure in GFRP strengthened beams. Further, the higher number of cracks in GFRP strengthened beams can be associated with the lower stiffness of GFRP bars as compared to steel bars, similar observations were made elsewhere^51,52^.

## Conclusions

This study presents an experimental investigation of the load–deflection response of beams with solid, hollow, prismatic, or non-prismatic sections. A total of six beams were tested under four-point monotonic bending. The following important conclusions can be drawn.

1. The intensity of shear was maximum in the case of prismatic section beams. The inclusion of a tapered section lowered the demand for shear
2. The initial stiffness of the beams was inversely related to the length of the tapered section along the beams.
3. The peak load was higher in solid section beams as compared to that hollow section beams. This difference in peak loads was prominent in prismatic beams.
4. Beams with non-prismatic sections were able to demonstrate higher energy dissipation capacities than beams with prismatic sections. Further, the energy dissipation capacities of beams with non-prismatic hollow sections were higher than beams with non-prismatic solid sections.
5. The adopted finite element modeling strategy resulted in close agreement with experimental crack patterns at ultimate failure. However, the ultimate failure loads predicted by nonlinear modeling were generally higher than their corresponding experimental results.
6. The results of the parametric study showed that the ultimate loads of non-prismatic solid and hollow sections RC beams were increased as the strength of concrete and reinforcement ratio of longitudinal steel bars were increased.
7. Beams strengthened with GFRP bars were able to demonstrate capacities that were comparable to that of steel-reinforced beams.

### Supplementary Information

Supplementary Information 1.

Supplementary Information 2.

Supplementary Information 3.

Supplementary Information 4.

Supplementary Information 5.

Supplementary Information 6.

Supplementary Information 7.

Supplementary Information 8.

Supplementary Information 9.

Supplementary Information 10.

Supplementary Information 11.

Supplementary Information 12.

Supplementary Information 13.

Supplementary Information 14.

Supplementary Information 15.

Supplementary Information 16.

## Data Availability

The datasets used and/or analysed during the current study are available from the corresponding author on reasonable request.
